# Prevalence and Risk Factors for Acute Kidney Injury in COVID-19-Hospitalized Patients in Poland Across Three Pandemic Periods

**DOI:** 10.3390/jcm14041384

**Published:** 2025-02-19

**Authors:** Paweł Edyko, Marta Zdunek, Maja Nowicka, Ilona Kurnatowska

**Affiliations:** 1 Student Scientific Society Affiliated with the Department of Internal Medicine and Transplant Nephrology, Medical University of Łódź, 90-419 Łódź, Poland; edyko.pawel@gmail.com (P.E.); marta.zdunek18@gmail.com (M.Z.); 2 Department of Internal Medicine and Transplant Nephrology, Medical University of Łódź, Kopcińskiego 22, 90-419 Łódź, Poland; maja.nowicka@umed.lodz.pl

**Keywords:** acute kidney injury, COVID-19 infection, prevalence, risk factor, outcome

## Abstract

**Background/Objectives**: Acute kidney injury (AKI) is a serious and prevalent complication of COVID-19. This study examines the prevalence, risk factors, and outcomes of AKI in hospitalized COVID-19 patients. **Methods**: We analyzed the data of 1223 adult COVID-19 hospitalized patients from a single district hospital during three pandemic periods: 3 November 2020–31 December 2020, 17 March 2021–8 May 2021, and 4 November 2021–21 February 2022. The analysis included demographic data, comorbidities, laboratory results, chest radiographs (CT lung scans), and outcomes. **Results**: We found an overall AKI incidence of 29.02%. AKI patients versus non-AKI ones were significantly older (median age 76.0 vs. 71.0, *p* < 0.001) and had more comorbidities, especially previous renal diseases, heart failure, coronary artery disease, and hypertension; they also significantly more often used diuretics, angiotensin receptor blockers (ARBs), and angiotensin-converting enzyme inhibitors (ACE-Is). AKI patients more frequently presented with abnormal CT lung scans and had higher white blood cell counts, lower lymphocytes percentages, higher C-reactive protein (CRP) levels, and lower platelet counts. They more often required oxygen therapy, more days of hospitalization, and had higher mortality rates. **Conclusions**: Older age, comorbidities, the use of diuretics, and renin-angiotensin system inhibitors (RASI) are key risk factors for AKI, which is consequently linked to a more severe disease course and poorer prognosis.

## 1. Introduction

The course of Severe Acute Respiratory Syndrome Coronavirus 2 (SARS-CoV-2) infection can range from asymptomatic to cold-like symptoms and progress to severe conditions [1]. In severe cases, patients may develop acute respiratory distress syndrome and experience multiorgan failure [2]. Furthermore, there is growing evidence indicating an increased incidence of AKI among patients affected by SARS-CoV-2 [3].

AKI is a sudden and rapidly progressing deterioration of kidney function, occurring within hours or days. The diagnosis is based on KDIGO criteria, demonstrated by changes in laboratory tests, such as increased serum creatinine or decreased urine output [4]. The occurrence of AKI in hospitalized patients is associated with an increased risk of chronic kidney disease (CKD) and an increased risk of death [3].

The pathophysiological mechanisms of AKI associated with COVID-19 may include a systemic inflammatory response with endothelial damage and vascular thrombosis but may also develop as a consequence of dehydration and hypotension or toxic effects of drugs and can lead to multiorgan failure [5]. The virus can also damage the renal parenchyma directly. The viral infiltration may target the renal tubular epithelium and podocytes. This intrusion sets forth a cascade of events encompassing mitochondrial dysfunction, the initiation of acute tubular necrosis, the induction of protein reabsorption vacuoles, the evolution of collapsing glomerulopathy, and the consequential leakage of proteins into Bowman’s capsule [6,7].

In response to COVID-19 infection and the subsequent development of complications, the angiotensin-converting enzyme 2 (ACE2) receptor may assume a significant role. ACE2 has been identified as a functional receptor for SARS-CoV-2 and is expressed across various organs, including the lungs, heart, intestines, and kidneys [8]. RASIs, recognized for their nephroprotective and cardioprotective properties, are commonly used since they are recommended as first-line treatments for conditions such as heart failure, hypertension, and the prevention of CKD progression [9].

The aim of the study was to conduct a retrospective analysis of the frequency and risk factors for AKI development and its consequence in patients hospitalized due to COVID-19 infection at a single-named hospital during three pandemic periods in Poland.

## 2. Materials and Methods

### 2.1. Population

We conducted a retrospective analysis of medical records from patients hospitalized with COVID-19 during three peak waves of the pandemic at the Regional Specialist Hospital in Lodz, a city with a population of 655,300, in central Poland [10]. Beginning in November 2021, the hospital was repurposed three times to specialize in treating COVID-19 patients: from 3 November 2020 to 31 December 2020 (W1); from 17 March 2021 to 8 May 2021 (W2); and from 4 November 2021 to 21 February 2022 (W3). The inclusion criteria for the analysis encompassed all adult patients who tested positive for COVID-19 via nasopharyngeal swab PCR, who were hospitalized in the infectious disease ward during waves W1, W2, and W3, and had at least two subsequent serum creatinine (sCr) measurements.

### 2.2. Analysis Data

Medical data, including demographics (gender and age), comorbidities, such as chronic obstructive pulmonary disease, asthma, pulmonary fibrosis, heart failure, hypertension, coronary artery disease, arrhythmias, diabetes, stroke or infarction, malignancies, venous thromboembolism, and previous kidney diseases, as well as laboratory assessments during hospitalization, were obtained from electronic hospital information systems. The following laboratory data on admissions were analyzed: sCr. urea, sodium, potassium and chlorine concentration, liver enzymes, CRP levels, blood morphology including white and red blood cell counts and platelet count, D-dimer levels, and blood gas analysis (pH, HCO_3_, BE-B). The sCr level from the day of admission or the following day was considered the baseline measurement. Additional data like CT lung scans, type of oxygen therapy, chronically taken medications, and time of onset of symptoms prior to hospital admission were also collected. The information regarding vaccination status and the types of vaccines administered, including Comirnaty (Pfizer-BioNTech, New York, NY, USA and Mainz, Germany), Spikevax (Moderna, Cambridge, MA, USA), Vaxzevria (AstraZeneca, Cambridge, UK), and COVID-19 Vaccine Janssen (Johnson & Johnson, New Brunswick, NJ, USA), was sourced from the patient government medical platform.

On admission, each patient was classified into the COVID-19 stage based on their blood gas saturation level. Patients whose oxygen saturation level was not below 95% were qualified as stage 1, those with saturation between 95 and 90% as stage 2, between 90 and 85% as stage 3, and patients with saturation below 85% as stage 4.

The diagnosis of the stage of AKI was based solely on sCr results obtained during hospitalization. AKI is defined as an increase or a decrease in sCr ≥ 0.3 mg/dL within 48 h, or an increase or decrease in sCr to ≥1.5 times baseline, which is known or presumed to have occurred within 7 days. Stage 1 is diagnosed if sCr increases or decreases to 1.5 to 1.9 times baseline, stage 2 if sCr increases or decreases to 2 to 2.9 times baseline, and when sCr increases or decreases by 3 times, patients are categorized as being stage 3 AKI [4,11,12,13]. eGFR was calculated using the CKD-EPI formula.

The analyzed data from AKI patients were compared to the data from patients with stable renal function during hospitalization (non-AKI group).

### 2.3. Statistical Analysis

Continuous data are presented as means with standard deviations (SDs) or as medians with the values of upper and lower quartiles, depending on the normality of distribution verified with the Shapiro–Wilk test, and were compared between the patient groups with an unpaired Student’s *t*-test or Mann–Whitney U test, depending on the normality of distribution. Ordinal data were compared with the Mann–Whitney U test. Nominal data are presented as numbers with percentages and were compared with the Chi2 test or with Fisher’s exact test depending on the number of individuals in subgroups. A multivariable logistic regression model with stepwise forward feature selection was used to investigate associations of baseline patients with the presence of AKI, including variables associated with outcomes in univariable analyses. *p* values lower than 0.05 were considered statistically significant. All analyses were performed with Microsoft Excel (Microsoft, Redmond, WA, USA) and with Statistica 13.0 software (StatSoft Polska, Kraków, Poland).

## 3. Results

Out of 1223 COVID-19-positive patients hospitalized during the three study periods, only three were excluded due to a lack of repeated laboratory tests. The overall incidence of AKI in the assessed population was 29.02% (*n* = 354), with 60.17% (*n* = 213) classified as stage 1, 26.84% (*n* = 95) as stage 2, and 12.99% (*n* = 46) as stage 3.

**Figure 1:**
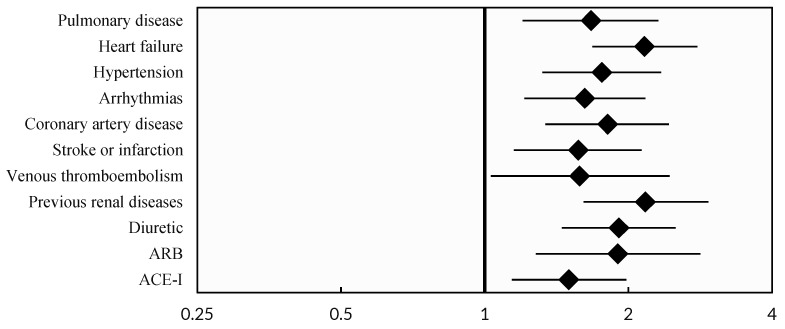
Forest Plot of Odds Ratios presenting significant factors from Table 1.

Table 1 (significant factors are illustrated in Figure 1) presents the patient demographic and baseline characteristics. Compared to non-AKI patients, those with AKI had a higher median age of 76 years (68, 84) versus non-AKI patients (71 years, 61, 82), *p* < 0.001, and a higher prevalence of comorbidities such as pulmonary disease, heart failure, hypertension, arrhythmias, coronary artery disease, stroke or infarction, venous thromboembolism, and previous kidney diseases. Diuretics, ARBs, and ACE-Is were significantly more commonly used in the AKI group.

AKI patients had a slightly higher severity of COVID-19, with 31.64% at level 3 compared to 24.94% for non-AKI patients. There were more changes in CT lung scans observed in AKI patients (89.6%) than in non-AKI ones (87.0%), *p* < 0.001.

AKI patients, compared to non-AKI ones, had lower median hemoglobin levels and higher median WBC counts with a lower percentage of lymphocytes and lower platelet counts at hospital admission. They also had higher levels of D-dimers, CRP, and aspartate transaminase activity. A worse baseline kidney function, indicated by higher serum creatinine and lower eGFR at admission, was a risk factor for AKI development during hospitalization. Laboratory results on hospital admission are presented in Table 2.

A higher percentage of AKI patients had received a complete vaccination against COVID-19 (20.9%) compared to non-AKI ones (15.7%), *p* = 0.220; 51.4% of patients with AKI had not been vaccinated compared to 57.04% of non-AKI ones (data presented in Table 3).

**Figure 2:**
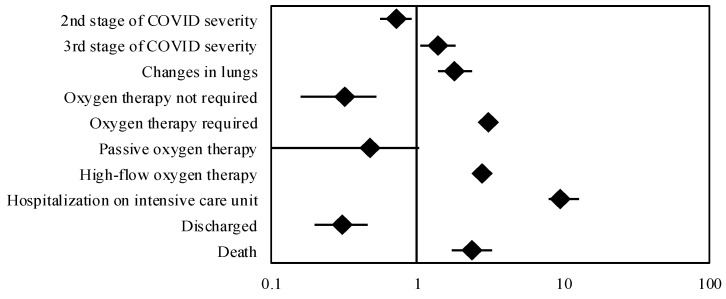
Forest Plot of Odds Ratios presenting significant factors from Table 1 and Table 4.

AKI, as compared to non-AKI patients, also more often required oxygen therapy (97.74% vs. 92.95%, *p* = 0.002), especially high-flow oxygen therapy (39.27% vs. 18.59%, *p* < 0.001), and intensive care unit (ICU) admission (29.38% vs. 4.16%, *p* < 0.001). They had longer hospital stays (17 days vs. 12 days, *p* < 0.001) and higher mortality rates (51.98% vs. 24.60%, *p* < 0.001). The details of the management and outcomes of COVID-19-hospitalized patients in relation to the occurrence of AKI are presented in Table 4 (significant factors are illustrated in Figure 2).

**Figure 3:**
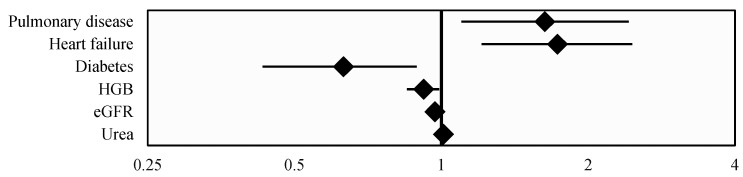
Forest Plot of Odds Ratios presenting significant factors from Table 5.

A multivariable logistic regression analysis identified the subsequent independent risk factors for AKI development in COVID-19-hospitalized patients: a history of pulmonary disease, heart failure, and diabetes mellitus, and the factors as indicated by laboratory tests results: the baseline kidney function including eGFR and urea concentration and hemoglobin level (Table 5 and Figure 3).

The data comparing the waves of the pandemic are presented in Table 6. Patients in the first wave were older than those in the second wave (80 vs. 76 years, *p* = 0.0019), with a slightly higher proportion of females in the first wave compared to the second (54.51% vs. 53.75%, *p* < 0.05) and a slightly smaller proportion in the third wave (54.51% vs. 54.57%, *p* < 0.05). The prevalence of AKI was higher in the third wave than in the second (33.2% vs. 21.6%, *p* = 0.0098), with more stage 1 AKI cases than in the second (64.5% vs. 56.5%, *p* = 0.0167).

## 4. Discussion

Our research confirmed that AKI is a frequent and serious complication in COVID-19-hospitalized patients [14]. The reported rates of AKI in COVID-19 patients varied across countries; our findings were similar to those in other European countries (29.0%) and the United States (27.3%) but were notably higher than those reported in China (9.6%) [15,16,17]. These differences may depend on the general characteristics of patients: the severity status of hospitalized patients, their comorbidities, the methods and criteria of diagnosing AKI, or the system of care. Our investigated population comprised exclusively COVID-19 patients who necessitated hospitalization, as well as internal medicine patients who were initially admitted to a different hospital and subsequently transferred to our institution upon the onset of COVID-19 infection.

Our findings aligned with previous observations that an older age is a significant risk factor for severe outcomes in COVID-19; therefore, an older mean age in our study population may have contributed to the higher incidence of AKI than in the UK and US cohorts, where the mean age was lower [16,18]. Interestingly, while other studies reported a higher proportion of men with COVID-19 developing AKI, our study did not find the male gender to be an independent risk factor [16,17].

We found that heart failure and previous renal diseases were the comorbidities most significantly associated with AKI development in COVID-19 patients. Such findings have been also reported in the literature [18,19]. Other studies have additionally identified hypertension and diabetes as significant AKI risk factors [19,20]. Our research confirmed those observations. Conditions like heart failure, coronary artery disease, hypertension, and diabetes often coexist with kidney dysfunction. In our study, they were also the risk factors for AKI development in COVID-19 patients.

We observed that SARS-CoV-2 infected patients who developed AKI, compared to non-AKI patients, exhibited specific laboratory abnormalities at admission, especially higher CRP levels, elevated D-dimer levels, elevated liver enzymes, the indicators of kidney function damage, as well as more pronounced metabolic acidosis, with lower bicarbonates and negative base excess and lower blood potassium levels. These observations are confirmed in the literature, where baseline elevated levels of sCr, urea, and CRP were most often detected in COVID-19 patients who developed AKI [20,21,22]. Elevated baseline levels of sCr and urea, in conjunction with the presence of metabolic acidosis observed in patients, may indicate the existence of CKD prior to admission and suggest that AKI was already present at the time of hospital admission. Our findings suggest that these parameters may play a crucial role in identifying high-risk patients and in predicting the course of the disease. Our observations were consistent with recent reports which indicated that renal damage in COVID-19 is linked to a heightened inflammation process, potentially contributing to extended and frequent ICU hospitalizations [23,24]. Moreover, the hematological differences observed in patients with AKI, both in our study and in the literature, involved elevated white blood cell counts, decreased lymphocyte counts, and lower platelet numbers, which may be considered early indicators or risk factors for AKI [17,25,26,27]. These findings suggest that certain alterations in blood cell profiles may be associated with the development of AKI and could potentially serve as early indicators or risk factors in the affected patients.

The association between ARBs, ACEIs, and diuretics with AKI is a compelling topic that has sparked considerable discussion in the medical community. These drugs are commonly prescribed for various conditions, including hypertension, heart failure, and CKD, and while they offer substantial benefits, they can also impact kidney function. During acute infections, these medications may increase the risk of kidney damage by exacerbating dehydration, reducing filtration pressure, and potentially inducing hypotension, particularly in febrile and critically ill patients [28]. The presence of dehydration and hypotension, especially in conjunction with elevated inflammatory markers, significantly increases the risk of developing AKI, particularly in elderly individuals [29]. In terms of mechanisms, angiotensin renin aldosterone inhibitors can affect the expression of the ACE2 enzyme, which also functions as a receptor for SARS-CoV-2, allowing the virus to penetrate host cells. Changes in ACE2 activity and expression could play a role in the infection and severity of COVID-19. However, the overall impact is ambiguous due to the complex effects of ACE2 [30]. In our study, the mentioned drug groups were statistically significantly more often used by patients who developed AKI, similar to a study conducted in France [22], but in contrast to studies conducted in the USA [31,32] and Italy [33]. Additionally, the critical role of diuretics has been highlighted in a previous study on the development of AKI in patients with COVID-19 [34]. Their classification as risk factors in the univariate model may be linked to the regular use of these medications in patients with CKD and associated comorbidities. This indicates that the observed morbidity is reflective more of the patient’s overall health rather than the inherent harmfulness of the drugs, as demonstrated by our multivariate model results. However, we cannot rule out the role of direct ACE2 receptor blockades in the severe course of infection including AKI development.

Numerous studies, including ours, have demonstrated that patients who develop AKI need extended hospital stays and require more frequent admissions to the ICU compared to those without AKI [20,35,36]. Moreover, AKI in our study was a risk factor of death, with a frequency comparable to other observations in the literature. Other research has also reported a nearly 50% mortality rate among SARS-CoV-2-infected patients who developed AKI [37,38,39].

Less than a quarter of our studied population had been vaccinated against COVID-19. The vaccination rates were comparable to those documented in a study focusing on COVID-related hospitalizations among American adults during a timeframe similar to that in our investigation [40]. Our study found that COVID vaccination did not act as a protective factor against AKI development, which is in agreement with Lukowsky et al.’s observations [41]. This can be explained by the fact that high-risk individuals including older patients with comorbidities were more frequently vaccinated and required hospitalization more often than vaccinated but younger people. In our study, an older age and high comorbidity were the key risk factors for AKI which may explain the lack of a noticeable protective role of vaccinations in preventing the development of AKI in our researched population.

Several limitations should be remembered when interpreting the results of our study. Our data were solely retrospectively extracted from electronic records; thus, there was absence and missing information on crucial aspects such as urine output, albuminuria incidence, blood pressure levels, hydration status, subsequent hospitalization occurrences, treatment specifics, and the potential impact of the treatment on AKI development. Moreover, we did not have knowledge about patients’ kidney function before admission.

## 5. Conclusions

Acute kidney injury is a common complication in patients with COVID-19, especially among elderly patients with comorbidities and those treated with diuretics and RASIs. AKI also led to a more severe disease course and more than doubled the risk of death. Considering this, further research to determine the most effective clinical approach in patients developing AKI during COVID-19 should be conducted.

## Figures and Tables

**Table 1:** Baseline characteristics and information on admission—univariate analysis of risk factors associated with acute kidney injury.

Number of Patients %, (*n*)	All Patients 100 (1220)	AKI 29.02 (354)	Non-AKI 70.98 (866)	*p*-Value	OR (95% Cl)
Age, median (IQR)	73 (63, 83)	76 (68, 84)	71 (61, 82)	*p* < 0.001	
Female, % (*n*)	54.34 (663)	55.08 (195)	54.04 (468)	*p* = 0.740	OR = 1.23 (0.75–1.23)
Male, % (*n*)	45.66 (557)	44.92 (354)	45.96 (398)	*p* = 0.740	OR = 1.23 (0.75–1.23)
BMI					
Median (IQR)	28.25 (24.76, 31,90)	29.03 (24.86, 32.28)	29.23 (24.67, 31.60)	*p* = 0.401	
No data, % (*n*)	38.28 (447)	47.46 (168)	34.53 (299)		
Comorbidities, % (*n*)					
Pulmonary disease	15.33 (187)	20.34 (72)	13.28 (115)	*p* = 0.002	OR = 1.67 (1.20–2.31)
Heart failure	35.57 (434)	48.59 (172)	30.25 (262)	*p* < 0.001	OR = 2.16 (1.68–2.79)
Hypertension	67.46 (823)	75.99 (269)	63.97 (554)	*p* < 0.001	OR = 1.76 (1.32–2.34)
Arrhythmias	20.82 (254)	26.84 (95)	18.36 (159)	*p* = 0.001	OR = 1.62 (1.21–2.17)
Coronary artery disease	19.67 (240)	26.84 (95)	16.74 (145)	*p* < 0.001	OR = 1.81 (1.34–2.43)
Diabetes	28.52 (348)	30.23 (107)	27.83 (241)	*p* = 0.441	OR = 1.11 (0.85–1.46)
Stroke or infarction	18.11 (221)	23.16 (82)	16.05 (139)	*p* = 0.004	OR = 1.57 (1.15–2.13)
Malignancies	15.49 (189)	15.54 (55)	15.47 (134)	*p* = 0.990	OR = 1.00 (0.71–1.40)
Venous thromboembolism	7.87 (96)	10.45 (37)	6.81 (59)	*p* = 0.035	OR = 1.58 (1.03–2.44)
Previous renal diseases	18.03 (220)	26.83 (95)	14.43 (125)	*p* < 0.001	OR = 2.17 (1.61–2.94)
Tobacco use	17.62 (215)	14.12 (50)	19.05 (165)	*p* = 0.106	OR = 0.75 (0.53–1.06)
Alcohol abuse	4.84 (59)	3.67 (13)	11.55 (46)	*p* = 0.325,	OR = 0.73 (0.39–1.37)
Medications, % (*n*)					
Diuretic	28.77 (351)	37.01 (131)	25.40 (220)	*p* < 0.001,	OR = 1.91 (1.45–2.51)
ARB	9.43 (115)	13.28 (47)	7.85 (68)	*p* = 0.001,	OR = 1.90 (1.28–2.83)
ACE-I	29.10 (355)	33.90 (120)	27.14 (235)	*p* = 0.004,	OR = 1.50 (1.14–1.98)
Other medications	0.33 (4)	0.28 (1)	0.35 (3)	*p* = 0.909,	OR = 0.88 (0.09–1.21)
COVID-19 severity, % (*n*)					
1	27.95 (341)	27.97 (99)	27.94 (242)	*p* = 0.964	OR = 1.01 (0.76–1.33)
2	42.70 (521)	37.01 (131)	45.03 (390)	*p* = 0.011	OR = 0.72 (0.56–0.93)
3	26.89 (328)	31.64 (112)	24.94 (216)	*p* = 0.015	OR = 1.40 (1.07–1.84)
4	1.31 (16)	1.98 (7)	1.04 (9)	*p* = 0.188	OR = 1.93 (0.71–5.22)
Changes on CT scan					
Changes in lungs, % (*n*)	87.0 (1062)	89.6 (317)	86.0 (745)	*p* < 0.001	OR = 1.83 (1.40–2.38)
Percentage of lungs occupied by lesions seen on CT scan, median (IQR)	25 (5, 45)	25 (10, 50)	22.5 (5, 40)	*p* = 0.257	
Other information					
Days from COVID-19 symptoms to admission, median (IQR)	3 (1, 6)	3 (1, 7)	3 (1, 6)	*p* = 0.406	

Abbreviations: ACE-I, angiotensin-converting enzyme inhibitor; ARB, angiotensin receptor blocker.

**Table 2:** Baseline laboratory results in SARS-CoV-2-infected patients.

Factor	All Patients	AKI	No AKI	*p*-Value
Laboratory Results on Admission, Median (IQR)				
HGB [g/dL]	13.6 (12.10, 14.80)	13.35 (11.80, 14.70)	13.8 (12.20, 14.80)	*p* = 0.060
WBC [×10^3^/μL]	7.1 (5.20, 10.20)	7.4 (5.20, 10.60)	7.1 (5.20, 9.80)	*p* = 0.002
LYMPH% [%]	12.9 (8.05, 19.50)	11.9 (7.70, 17.30)	13.45 (8.20, 20.40)	*p* = 0.002
PLT [×10^3^/μL]	187 (142, 243)	170 (130.00, 237.00)	192.5 (150.00, 249.00)	*p* = 0.002
Na [mmol/L]	137 (135.00, 140.00)	137 (134.00, 140.00)	137 (135.00, 140.00)	*p* = 0.883
K [mmol/L]	4.2 (3.70, 4.60)	4.2 (3.70, 4.60)	4.2 (3.80, 4.60)	*p* < 0.001
Cl [mmol/L]	100.1 (96.40, 103.20)	100.5 (96.50, 103.10)	100.05 (96.40, 103.20)	*p* = 0.244
D-dimer [μg/mL]	1.16 (0.73, 1.94)	1.39 (0.85, 2.30)	1.11 (0.69, 1.82)	*p* < 0.001
pH	7.44 (7.41, 7.48)	7.43 (7.39, 7.47)	7.45 (7.41, 7.48)	*p* < 0.001
HCO_3_-ST [mmol/L]	22.7 (21.10, 24.30)	21.8 (19.90, 23.90)	23 (21.70, 24.40)	*p* < 0.001
BE-B [mmol/L]	−2 (−3.90, −0.10)	−3.1 (−5.60, −0.70)	−1.7 (−3.40, 0.20)	*p* < 0.001
CRP [mmol/L]	59 (22, 126.20)	84.3 (41.70, 159.10)	51.3 (17.80, 107.70)	*p* < 0.001
ALT [U/L]	26.8 (17.80, 45.10)	26.3 (17.55, 43.85)	27.55 (17.80, 45.40)	*p* = 0.175
AST [U/L]	41.9 (26.90, 65.70)	45.05 (29.00, 70.55)	40.5 (25.70, 63.80)	*p* = 0.045
Renal function, median (IQR)				
Baseline SCr [mg/dL]	1.09 (0.84, 1.49)	1.36 (1.06, 1.89)	0.99 (0.76, 1.29)	*p* < 0.001
Baseline GFR [mL/min/1.73 m]	60.21 (40.24, 86.41)	45.95 (31.38, 64.47)	71.85 (49.92, 90.21)	*p* < 0.001
Baseline Urea [mg/dL]	45.90 (31.90, 74.30)	66.95 (45.20, 101.90)	40.20 (29.10, 56.50)	*p* < 0.001

Abbreviations: HGB, hemoglobin; WBC, white blood cell; LYMPH%, lymphocytes%; PLT, platelet count; CRP, C-reactive protein; ALT, alanine transaminase; AST, aspartate transferase; Baseline SCr, baseline serum creatinine; Baseline GFR, baseline glomerular filtration rate.

**Table 3:** Vaccination against COVID-19 in hospitalized SARS-CoV-2-infected patients with and without AKI.

Factor	All Patients	AKI	Non-AKI	*p*-Value	OR (95% Cl)
Vaccination Against SARS-CoV-2%, (*n*)					
Yes	24.73 (302)	15.75 (105)	22.74 (197)	*p* = 0.012	OR = 1.45 (1.08–1.96)
No	55.41 (676)	51.41 (182)	57.04 (494)		
Incomplete (1 dose)	3.93 (48)	1.95 (13)	4.04 (35)	*p* = 0.726	OR = 0.89 (0.46–1.71)
Complete (2 doses)	16.97 (207)	20.90 (74)	15.36 (133)	*p* = 0.220	OR = 1.46 (1.06–2.02)
Complete with reminder (3 doses)	2.79 (34)	3.95 (14)	2.09 (20)	*p* = 0.122	OR = 1.72 (0.86–3.46)
Reconvalescent	1.07 (13)	1.13 (4)	1.04 (9)		

**Table 4:** Management and outcomes of COVID-19-hospitalized patients according to the occurrence of acute kidney injury.

Factor	All Patients	Aki	Non-AKI	*p*-Value	OR (95% CI)
Oxygen Therapy					
Oxygen therapy required	94.34 (1151)	97.74 (346)	92.95 (801)	0.002	OR = 3.10 (1.47–6.57)
Passive oxygen therapy, % (*n*)	69.75 (851)	58.47 (207)	74.36 (644)	<0.001	OR = 0.48 (0.37–0.63)
High-flow oxygen therapy, % (*n*)	24.59 (300)	39.27 (139)	18.59 (161)	<0.001	OR = 2.83 (2.15–3.72)
Outcomes					
Hospitalization in intensive care unit (ICU), % (*n*)	11.48 (140)	29.38 (104)	4.16 (36)	<0.001	OR = 9.59 (6.40–14.37)
Period of hospitalization, days, median (IQR)	13 (9.19)	17 (11, 28)	12 (9, 16)	<0.001	
Discharged, % (*n*)	62.87 (767)	43.50 (154)	70.79 (613)	<0.001	OR = 0.31 (0.25–0.41)
Death, % (*n*)	32.54 (397)	51.98 (184)	24.60 (213)	<0.001	OR = 2.41 (1.87–3.12)
Transfer to another hospital, % (*n*)	4.59 (56)	4.52 (16)	4.62 (40)	0.940	OR = 0.98 (0.54–1.77)
Renal function, median (IQR)					
Discharged SCr [mg/dL]	0.88 (0.71, 1.22)	1.11 (0.78, 1.78)	0.82 (0.68, 1.04)	0.024	
Discharged GFR [mL/min/1.73 m]	80.64 (52.29, 95.10)	62.20 (32.00, 88.41)	85.95 (67.02, 98.12)	0.002	
Discharged Urea [mg/dL]	40.1 (27.83, 77.95)	65.55 (36.7, 128.43)	34 (26, 54.3)	0.001	

Abbreviations: Discharged SCr, discharged serum creatinine; Discharged GFR, discharged glomerular filtration rate.

**Table 5:** Multivariable logistic regression analysis of risk factors associated with acute kidney injury (only statistically significant factors).

Factor	Odds Ratios	*p*-Value
Comorbidities		
Pulmonary disease	1.63 (1.10, 2.42)	0.015
Heart failure	1.73 (1.21, 2.46)	0.002
Diabetes mellitus	0.63 (0.43, 0.89)	0.009
Laboratory results on admission to hospital		
Hemoglobin level	0.92 (0.85, 0.99)	0.019
Glomerular filtration rate (mL/min/1.73 m^2^)	0.97 (0.96, 0.98)	0.001
Urea level	1.01 (1.00, 1.01)	0.008

**Table 6:** Analysis of statistical differences between waves.

Factor	Wave	Wave 1	Wave 2	Wave 3
Age	Wave 1		*p* = 0.0019	*p* = 0.094
	Wave 2	*p* = 0.0019		*p* = 0.045
	Wave 3	*p* = 0.0944	*p* = 0.0457	
Gender	Wave 1		*p* < 0.05	*p* < 0.05
	Wave 2	*p* < 0.05		*p* = 1.000
	Wave 3	*p* < 0.05	*p* = 1.000	
AKI occurrence	Wave 1		*p* = 0.5856	*p* = 0.6353
	Wave 2	*p* = 0.5856		*p* = 0.0098
	Wave 3	*p* = 0.6353	*p* = 0.098	
Stage of AKI	Wave 1		*p* = 0.4757	*p* = 1.000
	Wave 2	*p* = 0.4757		*p* = 0.0167
	Wave 3	*p* = 1.000	*p* = 0.0167	
Deaths	Wave 1		*p* = 1.000	*p* = 1.000
	Wave 2	*p* = 1.000		*p* = 0.2614
	Wave 3	*p* = 1.000	*p* = 0.2614	

## Data Availability

Data are contained within the article.

## References

[1] Li M., Wang H., Tian L., Pang Z., Yang Q., Huang T., Fan J., Song L., Tong Y., Fan H. (2022). COVID-19 Vaccine Development: Milestones, Lessons and Prospects. Signal Transduct. Target. Ther..

[2] Ahmadian E., Hosseiniyan Khatibi S.M., Razi Soofiyani S., Abediazar S., Shoja M.M., Ardalan M., Zununi Vahed S. (2021). COVID-19 and Kidney Injury: Pathophysiology and Molecular Mechanisms. Rev. Med. Virol..

[3] Matsumoto K., Prowle J.R. (2022). COVID-19-Associated AKI. Curr. Opin. Crit. Care.

[4] KDIGO Acute Kidney Injury Work Group (2012). KDIGO Clinical Practice Guideline for Acute Kidney Injury. Kidney Int. Suppl..

[5] Mokhtari T., Hassani F., Ghaffari N., Ebrahimi B., Yarahmadi A., Hassanzadeh G. (2020). COVID-19 and Multiorgan Failure: A Narrative Review on Potential Mechanisms. J. Mol. Histol..

[6] Varga Z., Flammer A.J., Steiger P., Haberecker M., Andermatt R., Zinkernagel A.S., Mehra M.R., Schuepbach R.A., Ruschitzka F., Moch H. (2020). Endothelial Cell Infection and Endotheliitis in COVID-19. Lancet.

[7] Su H., Yang M., Wan C., Yi L.-X., Tang F., Zhu H.-Y., Yi F., Yang H.-C., Fogo A.B., Nie X. (2020). Renal Histopathological Analysis of 26 Postmortem Findings of Patients with COVID-19 in China. Kidney Int..

[8] Hassanein M., Radhakrishnan Y., Sedor J., Vachharajani T., Vachharajani V.T., Augustine J., Demirjian S., Thomas G. (2020). COVID-19 and the Kidney. Cleve. Clin. J. Med..

[9] Zhang Y., He D., Zhang W., Xing Y., Guo Y., Wang F., Jia J., Yan T., Liu Y., Lin S. (2020). ACE Inhibitor Benefit to Kidney and Cardiovascular Outcomes for Patients with Non-Dialysis Chronic Kidney Disease Stages 3–5: A Network Meta-Analysis of Randomised Clinical Trials. Drugs.

[10] Łódź Population Data. World Population Review. https://worldpopulationreview.com/cities/poland/lodz.

[11] Bellomo R., Ronco C., Kellum J.A., Mehta R.L., Palevsky P., Acute Dialysis Quality Initiative Workgroup (2004). Acute Renal Failure—Definition, Outcome Measures, Animal Models, Fluid Therapy, and Information Technology Needs: The Second International Consensus Conference of the Acute Dialysis Quality Initiative (ADQI) Group. Crit. Care.

[12] Yaqub S., Hashmi S., Kazmi M.K., Ali A.A., Dawood T., Sharif H. (2022). A Comparison of AKIN, KDIGO, and RIFLE Definitions to Diagnose Acute Kidney Injury and Predict the Outcomes after Cardiac Surgery in a South Asian Cohort. Cardiorenal Med..

[13] Wainstein M., MacDonald S., Fryer D., Young K., Balan V., Begum H., Burrell A., Citarella B.W., Cobb J.P., Kelly S. (2022). Use of an Extended KDIGO Definition to Diagnose Acute Kidney Injury in Patients with COVID-19: A Multinational Study Using the ISARIC–WHO Clinical Characterisation Protocol. PLoS Med..

[14] Głowacka M., Lipka S., Młynarska E., Franczyk B., Rysz J. (2021). Acute Kidney Injury in COVID-19. Int. J. Mol. Sci..

[15] Xu H., Garcia-Ptacek S., Annetorp M., Bruchfeld A., Cederholm T., Johnson P., Kivipelto M., Metzner C., Religa D., Eriksdotter M. (2021). Acute Kidney Injury and Mortality Risk in Older Adults with COVID-19. J. Nephrol..

[16] Zahid U., Ramachandran P., Spitalewitz S., Alasadi L., Chakraborti A., Azhar M., Mikhalina G., Sherazi A., Narh J.T., Khattar P. (2020). Acute Kidney Injury in COVID-19 Patients: An Inner City Hospital Experience and Policy Implications. Am. J. Nephrol..

[17] Tan L.S., Huang X.Y., Wang Y.F., Jia Y., Pang Q.L., Zhang W.X., Xiong Z.Y., Huang L., Li J.X. (2020). COVID-19-Related Renal Dysfunction: Pathophysiology and Interventions. Am. J. Transl. Res..

[18] Kolhe N.V., Fluck R.J., Selby N.M., Taal M.W. (2020). Acute Kidney Injury Associated with COVID-19: A Retrospective Cohort Study. PLoS Med..

[19] Hidayat A.A., Gunawan V.A., Iragama F.R., Alfiansyah R., Hertanto D.M., Tjempakasari A., Thaha M. (2023). Risk Factors and Clinical Characteristics of Acute Kidney Injury in Patients with COVID-19: A Systematic Review and Meta-Analysis. Pathophysiology.

[20] Jewell P.D., Bramham K., Galloway J., Post F., Norton S., Teo J., Fisher R., Saha R., Hutchings S., Hopkins P. (2021). COVID-19-Related Acute Kidney Injury: Incidence, Risk Factors and Outcomes in a Large UK Cohort. BMC Nephrol..

[21] Sabaghian T., Kharazmi A.B., Ansari A., Omidi F., Kazemi S.N., Hajikhani B., Vaziri-Harami R., Tajbakhsh A., Omidi S., Haddadi S. (2022). COVID-19 and Acute Kidney Injury: A Systematic Review. Front. Med..

[22] Anees M., Farooq O., Raza M., Mumtaz A. (2022). Frequency and Risk Factors for Acute Kidney Injury in Patients with COVID-19. Pak. J. Med. Sci..

[23] Hu B., Guo H., Zhou P., Shi Z.L. (2021). Characteristics of SARS-CoV-2 and COVID-19. Nat. Rev. Microbiol..

[24] Shrestha S., Zhang Y., Najafi W., Halik A., Chou J., Siu M.K.M., Dhillon M., Weisman D.S. (2024). Outcome Comparison in Hospitalized COVID-19 Patients with and without AKI. J. Community Hosp. Intern. Med. Perspect..

[25] Sinha S., Bansode J., Sayed S., Ahmad S., Swami R., Mehta K. (2022). Acute Kidney Injury in COVID-19: Clinical Profile and Outcome. Indian J. Nephrol..

[26] Fisher M., Neugarten J., Bellin E., Yunes M., Stahl L., Johns T.S., Abramowitz M.K., Levy R., Kumar N., Mokrzycki M.H. (2021). AKI in Hospitalized Patients with and without COVID-19: A Comparison Study. BMC Nephrol..

[27] Wang J., Wang Z., Zhu Y., Li H., Yuan X., Wang X., Wang Y., Hu J., Feng C., Liu C. (2020). Identify the Risk Factors of COVID-19-Related Acute Kidney Injury: A Single-Center, Retrospective Cohort Study. J. Clin. Med..

[28] Lea-Henry T.N., Baird-Gunning J., Petzel E., Roberts D.M. (2017). Medication Management on Sick Days. Aust. Prescr..

[29] Gameiro J., Fonseca J.A., Outerelo C., Lopes J.A. (2020). Acute Kidney Injury: From Diagnosis to Prevention and Treatment Strategies. J. Clin. Med..

[30] Ruilope L.M., Bakris G.L. (2020). Renin–Angiotensin–Aldosterone System Inhibitors in Patients with COVID-19. Lancet Respir. Med..

[31] Mehta N., Kalra A., Nowacki A.S., Anjewierden S., Han Z., Bhat P., Carmona-Rubio A.E., Jacob M., Procop G.W., Harrington S. (2020). Association of Use of Angiotensin-Converting Enzyme Inhibitors and Angiotensin II Receptor Blockers with Testing Positive for COVID-19. JAMA Cardiol..

[32] Reynolds H.R., Adhikari S., Pulgarin C., Troxel A.B., Iturrate E., Johnson S.B., Hausvater A., Newman J.D., Berger J.S., Bangalore S. (2020). Renin–Angiotensin–Aldosterone System Inhibitors and Risk of COVID-19. N. Engl. J. Med..

[33] Mancia G., Rea F., Ludergnani M., Apolone G., Corrao G. (2020). Renin–Angiotensin–Aldosterone System Blockers and the Risk of COVID-19. N. Engl. J. Med..

[34] Zhang J., Pang Q., Zhou T., Meng J., Dong X., Wang Z., Zhang A. (2023). Risk Factors for Acute Kidney Injury in COVID-19 Patients: An Updated Systematic Review and Meta-Analysis. Ren. Fail..

[35] Diebold M., Zimmermann T., Dickenmann M., Schaub S., Bassetti S., Tschudin-Sutter S., Bingisser R., Heim C., Siegemund M., Osswald S. (2021). Comparison of Acute Kidney Injury in Patients with COVID-19 and Other Respiratory Infections: A Prospective Cohort Study. J. Clin. Med..

[36] Kanbay M., Medetalibeyoglu A., Kanbay A., Cevik E., Tanriover C., Baygul A., Şenkal N., Konyaoglu H., Akpinar T.S., Kose M. (2022). Acute Kidney Injury in Hospitalized COVID-19 Patients. Int. Urol. Nephrol..

[37] Małyszko J., Małyszko J.S., Wojtaszek E. (2024). Acute Kidney Injury in Hospitalized Patients with COVID-19. Pol. Arch. Intern. Med..

[38] Chávez-Íñiguez J.S., Cano-Cervantes J.H., Maggiani-Aguilera P., Lavelle-Góngora N., Marcial-Meza J., Camacho-Murillo E.P., Moreno-González C., Tanaka-Gutiérrez J.A., Zaragoza A.P.V., Rincón-Souza K.E. (2021). Mortality and Evolution Between Community- and Hospital-Acquired COVID-AKI. PLoS ONE.

[39] Chan L., Chaudhary K., Saha A., Chauhan K., Vaid A., Zhao S., Paranjpe I., Somani S., Richter F., Miotto R. (2021). AKI in Hospitalized Patients with COVID-19. J. Am. Soc. Nephrol..

[40] Havers F.P., Pham H., Taylor C.A., Whitaker M., Patel K., Anglin O., Kambhampati A.K., Milucky J., Zell E., Moline H.L. (2022). COVID-19-Associated Hospitalizations Among Vaccinated and Unvaccinated Adults 18 Years or Older in 13 US States, January 2021 to April 2022. JAMA Intern. Med..

[41] Lukowsky L.R., Der-Martirosian C., Northcraft H., Kalantar-Zadeh K., Goldfarb D.S., Dobalian A. (2024). Predictors of Acute Kidney Injury (AKI) Among COVID-19 Patients at the US Department of Veterans Affairs: The Important Role of COVID-19 Vaccinations. Vaccines.

